# Editorial: Strategies to fight exercise intolerance in neuromuscular disorders, volume II

**DOI:** 10.3389/fphys.2023.1229040

**Published:** 2023-06-07

**Authors:** Francesca Lanfranconi, Lucio Tremolizzo, Mauro Marzorati, Giuseppe D’Antona

**Affiliations:** ^1^ Centro Maria Letizia Verga, Fondazione Monza e Brianza per il Bambino e la sua Mamma, Monza, Italy; ^2^ Neurology Unit, Fondazione IRCCS San Gerardo dei Tintori, University of Milano-Bicocca, Monza, Italy; ^3^ Institute of Biomedical Technologies, National Research Council, Segrate (MI), Italy; ^4^ Centro di Ricerca Interdipartimentale nelle Attivitá Motorie e Sportive (CRIAMS)—Sport Medicine Centre, University of Pavia, Voghera, Italy

**Keywords:** exercise, diet, motor neuron disease, neuromuscular degenerative diseases, glycogen storage diseases, disability, inclusion

We are delighted to be Editors of the second volume of this Research Topic. In the first Research Topic, our conclusion focused on the need for a new cultural approach among clinicians and researchers to the use of exercise and diet as a safe and appropriate ways to fight neuromuscular diseases (NMD), in order to provide suitable care for children and adults (Lanfranconi et al., 2020). We can again raise our voice to other researchers who want to fill the knowledge gap, from cellular mechanisms to system disorders, and find concrete caretaking solutions for patients with NMD, in a strategy of constant close crosstalk between clinicians and exercise physiologists.

Overall, although some progress has been made in recent years, people with NMD continue to die earlier, have poorer health and experience more limitations in everyday functioning than others. These outcomes are also due to inequities faced by people with disabilities in all aspects of life, including the health system itself and access to physical activity programmes. Indeed, the lack of equity for people with motor disabilities is a matter of concern for authorities as stated by the World Health Organization (2022) Global report on health equity for persons with disability,” and a source of great frustration for the individual and their family.

As is well known, the aetiology of NMD can be inherited or acquired, acute and transient or chronic, degenerative or non-degenerative. The spectrum of clinical manifestations is very broad, but inevitably results in reduced exercise tolerance (Lanfranconi et al., 2020), impaired skeletal muscle oxidative metabolism (McDonald et al., 1995) and weakness (van der Kooi et al., 2005). The ultimate tragic outcome is an extraordinary challenge to the resilience of those affected, from childhood to adulthood and into the last part of their lives.

Precision-based exercise programmes for medically fragile individuals, such as those with NMD, are an increasing opportunity even in complex clinical settings where children, adolescents and adults are cared for (Stefanetti et al., 2020). The resulting physical inactivity is highly detrimental and can negatively affect whole-body homeostasis, leading to a potential downward spiral of the disease, thus contributing to a further reduction in the patient’s quality of life and social and occupational integration (Voet, 2019).

The impact of different types of diets on NMD is equally appealing because it affects mitochondrial function, oxidative stress, neuronal apoptosis, neuroinflammation and the microbiota-gut-brain axis (Tao et al., 2022). Although the full mechanisms of diet in the treatment of NMD remain to be elucidated, its clinical efficacy is attracting many new researchers. Diet, like precision-based exercise, is a good candidate for care.

We believe that precision-based exercise programmes and diet should be considered as one of the most important cornerstones of complex multimodal interventions aimed at preventing NMD and frailty where possible. In particular, precision-based training programmes and diet can be a powerful approach to counteract the conditions that threaten oxidative metabolism and loss of skeletal muscle function. In addition, the assessment of physiological responses to exercise and diet appears to be a powerful translational diagnostic tool that can extend the frontiers of human physiology into the clinical setting and extend the knowledge of clinical outcomes beyond resting conditions.

Certainly, accurate determination of individual exercise tolerance is a fundamental part of precision-based interventions. Voet et al. investigated the correlations between the two ventilatory (VT) and surface electromyography (sEMG) thresholds (Th1 and Th2), which are closely related during incremental exercise in healthy controls, in a cohort of NMD patients. They found that sEMG Th1 and Th2 occurred at relatively lower power levels than VTs in patients compared to healthy controls (most pronounced for sEMG Th1). Considering that muscle fibre type distribution is a fundamental determinant of sEMG Th1 and Th2, a fibre type shift towards a faster phenotype due to concomitant disuse is hypothesised as a possible contributor to this variation. Based on these findings, the authors suggest that in patients with NMD, differences in fiber type composition may contribute to muscle-specific strength and sEMG measurements during submaximal dynamic exercise are needed to characterise daily activities and to prescribe and evaluate rehabilitation interventions.

The same concept of having the right amount of exercise is applied to McArdle patients by Salazar-Martinez et al. An increase in the workload that induces the second wind phenomenon (i.e., an initial period of marked intolerance to dynamic exercise followed by a second phase of improved exercise tolerance) would potentially translate into an improvement in patients’ exercise tolerance in daily life. This means that higher aerobic fitness and an active lifestyle are associated with a higher workload that induces the second wind phenomenon, which has a positive effect on activities of daily living.

Another point was raised by Negro et al. when they tried to clarify the biochemical mechanisms related to nutrition and the physiological aspects of muscle metabolism related to exercise, in order to propose new theoretical bases of treatment which, if properly tested and validated by future trials, could be applied to improve the quality of life of patients affected by carnitine palmitoyltransferase II (CPTII) deficiency. As a matter of fact, this approach, that includes high-intensity intermittent exercise, has been already successfully used at least in a single case study of this disease (Parimbelli et al., 2021). Again, although there has been remarkable scientific progress in genetic analysis, pathophysiology and diagnosis, the current recommendations are still to follow a carbohydrate-rich diet with limited fat intake and to reduce or even eliminate exercise, without taking into account the long-term consequences of this approach.

The scoping review by Pedersen et al., addresses the results of strength training in patients with polyneuropathy. Although several studies indicate that strength training may be beneficial for these patients, no recommendation can yet be made due to the low methodological strength of most of the studies. The limited number of reports and their relatively low quality, using different functional outcomes, underline the importance of further studies to evaluate the effect of strength training on relevant functional and clinical outcomes.

## Conclusion

Precision-based exercise programmes and diet can be a powerful therapeutic approach, but as with any drug, when and the exact type and dose to be administered is matter of research.

The wall that needs to be broken today is the idea that NMD patients should not exercise at all: adaptation is the world to be used, not avoidance, and rigorously defined workloads are the key (Ferri et al., 2019).
